# Risk of venous thromboembolism following human papillomavirus vaccination: a systematic review and meta-analysis

**DOI:** 10.1016/j.gore.2026.102095

**Published:** 2026-04-26

**Authors:** Yushu Wang, Guangyu Ao, Claude Hughes

**Affiliations:** a IQVIA (Shanghai) Pharmaceutical Technology Co., Ltd., Chendu Branch, China; b Department of Nephrology, The First People’s Hospital of Chengdu, Chengdu, China; c Reproductive Health Center of Excellence and Therapeutic Science and Strategy Unit, IQVIA & Department of OB‑GYN, Duke University Medical Center, Durham, NC, USA

**Keywords:** Human papillomavirus vaccine, Venous thromboembolism, Safety, Meta-analysis

## Abstract

**Background:**

Sporadic reports linking human papillomavirus (HPV) vaccination to venous thromboembolism (VTE) have raised safety concerns. We intended to provide an updated and comprehensive evaluation of this potential association using recent large-scale data.

**Methods:**

We conducted a systematic review and *meta*-analysis of observational studies evaluating HPV vaccination and the risk of VTE. Major databases were searched from their inception through December 10, 2025. We pooled risk estimates using a random-effects model and assessed risk of bias using the ROBINS-I tool.

**Results:**

Eight observational studies involving millions of participants were included. The pooled analysis showed no statistically significant association between HPV vaccination and VTE risk (Risk Ratio = 0.87; 95%CI: 0.71–1.07; P = 0.183). Consistency was observed across all subgroups, including age (<18 vs. ≥18 years), sex (female vs. male), and vaccine valency (including 9-valent).

**Conclusion:**

Current evidence indicates that HPV vaccination is not associated with an increased risk of VTE. These findings reinforce the favorable safety profile of HPV vaccines across diverse populations and support continued vaccination programs.

## Highlights

• Past reports suggested that risk of venous thromboembolism (VTE) was increased after human papillomavirus (HPV) vaccination.
• We conducted a systematic review and *meta*-analysis of eight observational studies involving millions of participants.
• Current evidence indicates that HPV vaccination is not associated with an increased risk of VTE.

## Graphical abstract

**Figure:**
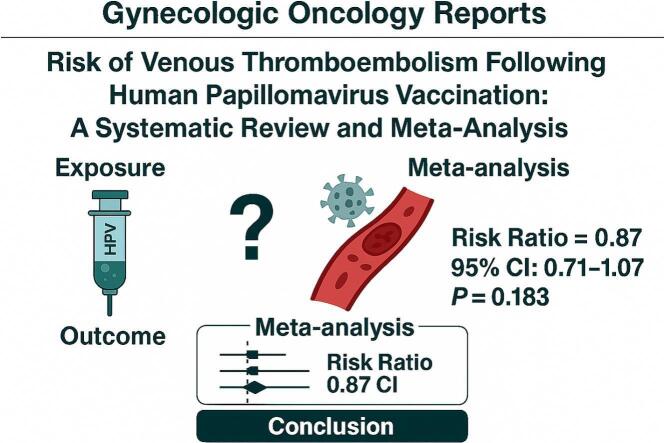
Our analysis indicates that HPV vaccination is not associated with an increased risk of VTE.

## 1 Introduction

Human papillomavirus (HPV) infection is a major global public health burden and is the primary cause of cervical cancer (de Martel et al., 2017). It is also a significant contributor to other malignancies, including anogenital and oropharyngeal cancers. Currently, prophylactic vaccination is established as the most effective strategy for preventing HPV-related diseases and has been included in routine immunization programs worldwide (WHO, 2022). However, despite the proven benefits of vaccination, maintaining high coverage rates is often challenged by public concerns regarding safety. In recent years, as vaccination programs have expanded to broader populations, ensuring public confidence in vaccine safety has become increasingly critical.

Among the potential adverse events following immunization, venous thromboembolism (VTE), which includes deep vein thrombosis (DVT) and pulmonary embolism (PE), has attracted particular attention due to its potential severity and clinical consequences. Although pre-licensure clinical trials demonstrated a favorable safety profile for HPV vaccines, these trials were typically limited by small sample size and follow-up duration (Moreira et al., 2016). Therefore, they were often insufficient to detect rare adverse events such as VTE. Consequently, sporadic reports from post-marketing surveillance have linked HPV vaccination to thrombotic events. These reports, even if rare, have perpetuated safety concerns among the public and contributed to vaccine hesitancy (Drolet et al., 2019).

Investigating the potential association between HPV vaccination and VTE has been challenging, and existing evidence remains controversial. Post-licensure observational studies have yielded conflicting findings. Some early signal detection analyses suggested a potential temporal association in adolescent females aged 9–17 years. In the Vaccine Safety Datalink study, a non-statistically significant elevated VTE risk (RR = 1.98) was observed in this age group after HPV4 vaccination; however, only five confirmed cases were identified, and all had established thrombotic risk factors (Gee et al., 2011). By contrast, subsequent large-scale investigations generally found no evidence of an increased risk (Arnheim-Dahlström et al., 2013, Naleway et al., 2016, Scheller et al., 2014). Furthermore, previous studies have predominantly focused on general safety profiles or autoimmune conditions, often assessing VTE only as a secondary outcome (Faksová et al., 2024, Yoon et al., 2021). As consistent and definitive data are not available, a clear conclusion regarding the thrombotic risk of HPV vaccination has not yet been reached. Given the rarity of VTE and the inconsistencies across individual studies, a dedicated quantitative synthesis is critically needed to resolve this uncertainty.

Currently, there is a lack of systematic reviews and *meta*-analyses specifically dedicated to quantifying the risk of VTE following HPV vaccination, particularly those incorporating the most recent large-scale data. With the accumulation of clinical evidence from robust study designs in recent years (Faksová et al., 2024, Yoon et al., 2021, Frisch et al., 2018), it is essential to re-evaluate this issue comprehensively. Thus, we conducted this systematic review and *meta*-analysis to determine whether HPV vaccination is associated with an increased risk of VTE. Additionally, we explored potential variations in risk across different demographic groups and vaccine types. This study aims to provide a robust and updated safety assessment to inform clinical practice and public health policy.

## 2 Methods

A comprehensive systematic review and *meta*-analysis was undertaken in accordance with the guidelines of the Preferred Reporting Items for Systematic Reviews and Meta-Analyses (PRISMA) of 2020 (Page et al., 2021) and Meta-analysis of Observational Studies in Epidemiology (MOOSE) guidelines (Stroup et al., 2000). The protocol for this *meta*-analysis is registered with PROSPERO (registration number: CRD420251243121).

### 2.1 Eligibility criteria

The exposure of interest was receipt of any prophylactic HPV vaccine, and the outcome was incident VTE, including DVT and/or PE. Studies that met the following conditions were included: (1) observational studies employing a cohort or self‑controlled design, including self-controlled case series and self-controlled risk interval studies or studies utilizing randomized controlled trial (RCT) design; (2) HPV vaccination exposure; (3) controls were individuals who did not receive an HPV vaccination; (4) the outcome of interest was the risk for VTE associated with HPV vaccination; (5) reported either a risk ratio (RR), hazard ratio (HR), odds ratio (OR), or incidence rate ratio (IRR) with 95% confidence intervals (CIs). Studies were excluded based on the following criteria: case reports or uncontrolled/non-comparative case series, non-human research, editorials, and review articles. Initially, titles and abstracts of identified articles were screened, followed by the acquisition and eligibility assessment of the full texts of articles considered potentially relevant. Two authors (YW and GA) independently conducted study selection, with any discrepancies resolved through discussion involving a senior author.

### 2.2 Search strategy

A comprehensive systematic search of PubMed, Embase, and the Cochrane Library databases was conducted from their inception to December 10, 2025. The search strategy employed both keywords and MeSH terms, including “Papillomavirus Vaccines,” “HPV vaccine,” “Human Papillomavirus,” “Gardasil,” “Cervarix,” or “9-valent,” combined with “Venous Thromboembolism,” “Venous Thrombosis,” “Pulmonary Embolism,” “Deep Vein Thrombosis,” “DVT,” or “PE.” The search was unrestricted by language or publication year. Additionally, the reference lists of the retrieved articles and relevant reviews were manually screened to identify additional studies.

### 2.3 Data extraction and quality assessment

Data extraction from the included studies was performed independently by two authors (YW and GA) using an electronic structured extraction form. Any discrepancies encountered were resolved by consulting a third author. The extracted data, standardized for consistency, included the following details: name of the first author, year of publication, sample size, country of study, study design, study period, observation windows, participant characteristics, vaccine valency, and diagnostic criteria for VTE. Quasi-experimental studies were evaluated using the Risk of Bias in Nonrandomized Studies-of Interventions tool (ROBINS-I), which addresses seven key domains: (1) bias due to confounding; (2) bias in selection of participants into the study; (3) bias in classification of interventions; (4) bias due to deviations from intended interventions; (5) bias due to missing data; (6) bias in measurement of outcomes; and (7) bias in selection of the reported results. Based on the overall assessment using the ROBINS-I tool, each quasi-experimental study was classified as having either a low risk of bias, moderate risk of bias, serious risk of bias, critical risk of bias, or lacking sufficient information to make a determination regarding the risk of bias (Sterne et al., 2016). YW and GA independently evaluated the methodological quality of each included study. Any disagreements were addressed and resolved through discussion between the two reviewers.

### 2.4 Statistical analysis

The pooled results were represented as the RR for VTE associated with HPV vaccination compared to no vaccination, with RRs serving as approximations for HRs or IRRs. Whenever feasible, we extracted adjusted effect estimates, along with their standard errors, for the association between HPV vaccination exposure and the outcome measures. If adjusted estimates were unavailable, we calculated the unadjusted risk ratio using raw data. The pooled RR with a 95% CI for VTE was determined using a random-effects model *meta*-analysis, given the anticipated clinical heterogeneity across the included studies. Heterogeneity was evaluated utilizing the Cochran Q-statistic and I^2^ tests, with statistical significance determined by a P value of less than 0.05. To explore potential sources of heterogeneity, subgroup analyses were stratified according to age, gender, study design, country, and vaccine valency. To assess the influence of individual studies, we conducted a leave-one-out sensitivity analysis by sequentially excluding each study and recalculating the pooled estimate. Publication bias was intended to be assessed using funnel plots if an outcome included 10 or more studies; however, since fewer than 10 studies were included, a funnel plot was not generated (Page et al., 2024). All *meta*-analyses were performed using the statistical software STATA version 15.0 (Stata Corporation, University of Texas, TX, USA).

## 3 Results

### 3.1 Literature searches and characteristics of included studies

**Fig. 1:**
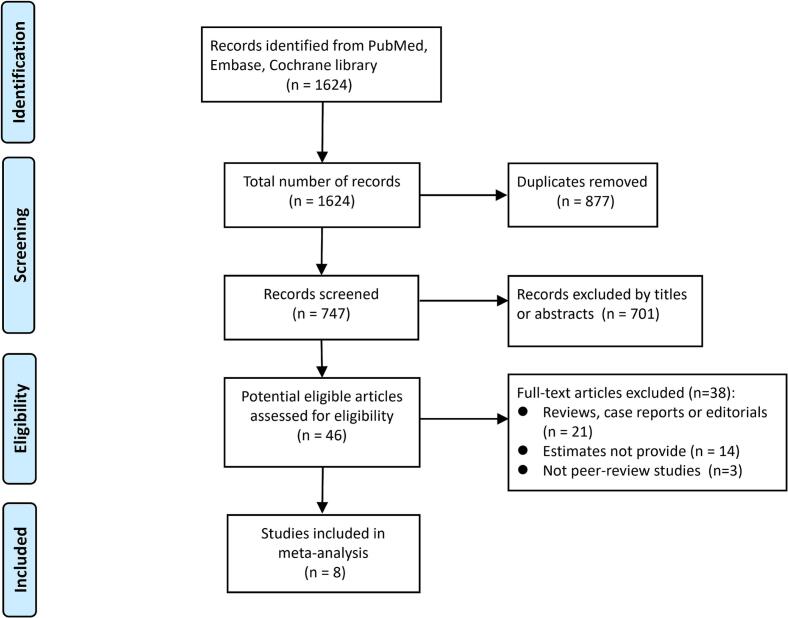
Flow diagram of selection process for inclusion of studies in *meta*-analysis.

As depicted in Fig. 1, the database searches yielded 1624 records. After de-duplication, 747 unique records underwent title and abstract screening, of which 701 were excluded. Forty-six full-text articles were assessed for eligibility; applying the prespecified exclusion criteria, eight studies were included (Gee et al., 2011, Arnheim-Dahlström et al., 2013, Scheller et al., 2014, Naleway et al., 2016, Faksová et al., 2024, Yoon et al., 2021, Frisch et al., 2018, Yih et al., 2016).

**Fig. 2:**
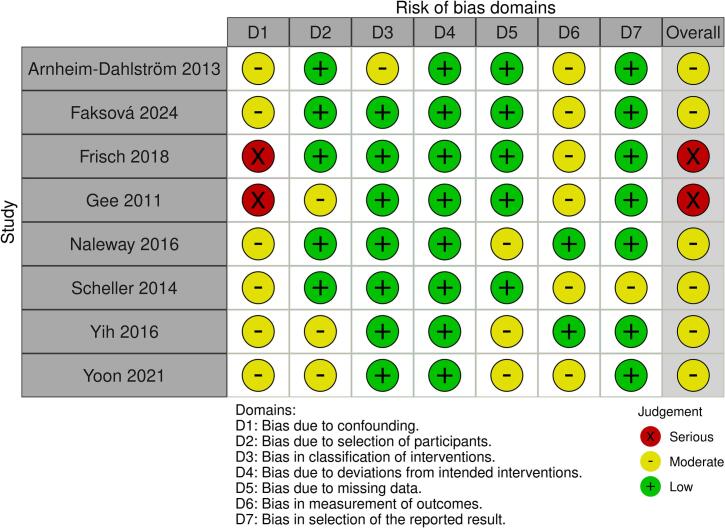
Risk of bias of included studies using ROBINS-I.

The characteristics of the included studies are summarized in Table 1. Among studies reporting a source population, participant counts ranged from 441,399 to 1,613,798, vaccinated/exposed sample sizes ranged from 7,384 to 650,737, and total administered doses ranged from 16,929 to 1,423,399. VTE was rare across included studies: cohort studies reported small numbers of events per person-time in exposed and unexposed/reference groups, and self-controlled analyses similarly observed limited case counts within prespecified post-vaccination risk windows relative to reference/control periods. Four studies were conducted in Denmark, three in the United States, and one in South Korea. With respect to study design, four were cohort studies and four were self-controlled designs (three self-controlled case series and one self-controlled risk interval); no randomized controlled trials met the inclusion criteria. The included publications spanned 2011–2024, while the underlying study periods collectively cover approximately 18 years (2006–2024). Study populations primarily comprised adolescents aged 10–26 years and were largely female, although some studies also included male participants. Specifically, Gee et al. provided age-stratified estimates, which allowed us to include two distinct effect estimates corresponding to (a) adolescents aged 9–17 years and (b) young adults aged 18–26 years. Faksová et al. reported estimates separately for females and males; accordingly, we entered these as two sex-specific records (Faksová 2024a for females and Faksová 2024b for males). Vaccine formulations evaluated were mainly quadrivalent HPV (HPV4), with fewer studies assessing nonavalent (HPV9) and bivalent (HPV2) vaccines. Outcome ascertainment relied on ICD coding (ICD-9 or ICD-10), with several studies supplementing case identification through medical record review or prescription data. Risk-of-bias appraisal classified two studies as having a serious risk of bias and six as having a moderate risk of bias (Fig. 2).

**Table 1:** Characteristics of included studies.

Study, Year	Country	Study Design	Study Period	Age (years)	Study population (n)	Vaccinated/Exposed (n)	Total vaccine doses administered (n)	Cohort: VTE Events/PY (Exposed) SCCS/SCRI:cases in risk period (n)	Cohort: VTE Events/PY (Unexposed) SCCS/SCRI: cases in reference period (n)	Sex (study population)	Vaccine Type	Follow-up Time (Risk Window)	Outcome Assessment
Gee 2011	USA	Cohort (historical)	2006–2009	Gee 2011a: youth (9–17 years); Gee 2011b: adult (18–26)	NR	NR	600,558	Observed cases within risk window: 8 (youth), 11 (adult)	Expected cases (historical): 4.04 (youth), 15.00 (adult)	Female	HPV4	1–42 days	ICD-9 codes, medical record review
Arnheim-Dahlström 2013	Denmark & Sweden	Cohort	2006–2010	10–17	997,585	296,826	696,420	21 / 149,817 PY	297 / 2,373,786 PY	Female	HPV4	0–90 days	ICD-10 codes
Scheller 2014	Denmark	SCCS	2006–2013	10–44	1,613,798	500,345	NR	29 cases (risk period)	860 cases (reference period)	Female	HPV4	1–42 days	ICD-10 codes, anticoagulant prescription
Naleway 2016	USA	SCCS	2008–2011	9–26	650,737	650,737	1,240,000	16 cases (risk period)	140 cases (unexposed observation time)	Female, Male	HPV4	1–60 days	ICD-9 codes, medical record review
Yih 2016	USA	SCRI	2006–2013	9–26	NR	650,475	1,423,399	13 cases (risk interval)	17 cases (control interval)	Female	HPV4	1–28 days	ICD-9 codes, medical record review
Frisch 2018	Denmark	Cohort	2006–2016	10–17	568,410	7,384	16,929	4 / 24,130 PY	657 / 4,333,875 PY	Male	HPV4	0–180 days and ≥ 181 days	ICD-10 codes
Yoon 2021	South Korea	Cohort	2017–2019	11–14	441,399	382,020	429,377	26 / 408,340 PY	6 / 60,628 PY	Female	HPV2 & HPV4	1 year	ICD-10 codes
Faksová 2024	Denmark	SCCS	2017–2022	10–17	854,586	350,687	673,530	Female: 3 cases (risk period) Male: <3 cases (risk period)	Female: 38 cases (reference period) Male: 25 cases (reference period)	Faksová 2024a: Female; Faksová 2024b: Male	HPV9	1–28 days	ICD-10 codes

SCCS: self-controlled case series; SCRI: self-controlled risk interval; VTE: venous thromboembolism; PY: person-years. NR: not reported.

### 3.2 Association between HPV vaccination and risk of VTE and subgroup analyses

**Fig. 3:**
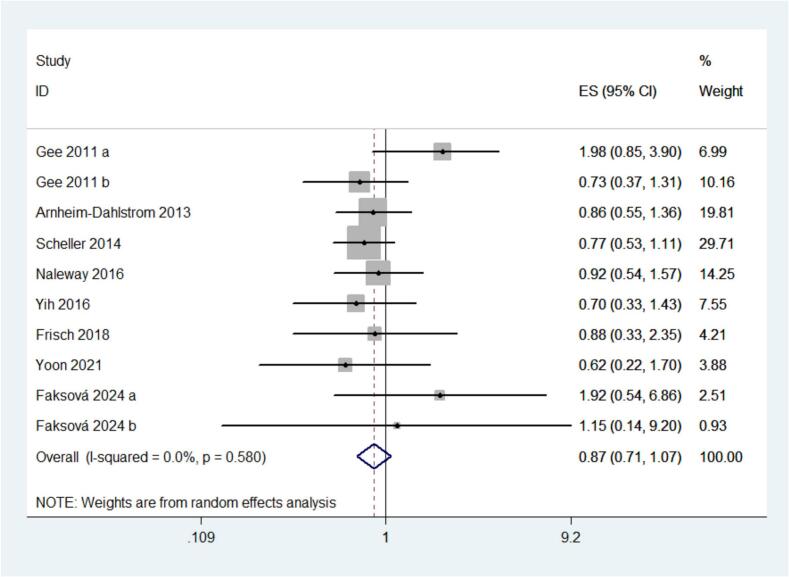
Risk of venous thromboembolism in participants receiving HPV vaccination

Fig. 3 presents the pooled association between human papillomavirus (HPV) vaccination and VTE risk, showing no statistically significant relationship (RR = 0.87; 95% CI, 0.71–1.07; I^2^ = 0%; P = 0.183). Prespecified subgroup analyses were conducted by age, sex, vaccine valency, study design, and country (Table 2). By age, no association was observed in either subgroup: <18 years and ≥18 years. By gender, estimates likewise indicated no association in females or males. By vaccine valency, results were non-significant for HPV4 and HPV9. By study design, neither cohort studies nor self-controlled case series designs demonstrated an association. Finally, by country, estimates from the USA and from other countries were similarly not statistically significant. Collectively, the subgroup findings are consistent with the overall absence of a statistically significant association between HPV vaccination and VTE. Sensitivity analysis by removing each study one at a time from the *meta*-analysis did not affect the overall results, supporting the robustness of the findings.

**Table 2:** Subgroup analyses of the association between HPV vaccination and venous thromboembolism.

Subgroup factor	Subgroup	RR	95% CI	I^2^	P value
Age	<18 years	1.05	0.78–1.42	0	0.737
	≥18 years	0.83	0.57–1.21	0	0.336
Gender	Female	0.88	0.71–1.09	7.2	0.249
	Male	0.92	0.38–2.25	0	0.861
Vaccine valency	HPV4	0.83	0.67–1.03	0	0.089
	HPV9	1.67	0.56–4.96	0	0.354
Study design	Cohort studies	0.93	0.65–1.32	21.1	0.677
	Self-controlled studies	0.84	0.64–1.10	0	0.195
Country	USA	0.96	0.63–1.45	39	0.833
	Non-USA	0.83	0.64–1.07	0	0.157

## 4 Discussion

In this study, we conducted a systematic review and *meta*-analysis to clarify the potential association between HPV vaccination and venous thromboembolism (VTE) using the most recent evidence. Our results indicated that HPV vaccination was not associated with an increased risk of VTE. Importantly, this lack of association was consistent across all subgroups, including different sexes (male and female), age groups (adolescents and young adults), and vaccine types (bivalent, quadrivalent, and nonavalent). Our study provides robust evidence supporting the safety of current HPV vaccination programs.

The safety of HPV vaccines has been a subject of controversy. Previous early surveillance studies, such as the analysis from the Vaccine Safety Datalink (VSD), raised concern mainly in females aged 9–17 years, in whom a non-significant elevated VTE risk was observed after HPV4 vaccination. However, this signal was based on only a small number of confirmed cases, all of whom had known thrombotic risk factors. Importantly, our *meta*-analysis directly addressed these clinically relevant strata through prespecified subgroup analyses by age (<18 vs ≥18 years) and sex, and no increased VTE risk was observed in either adolescents or females. These findings are consistent with subsequent large-scale cohort studies conducted in the US and Europe, which reported no significant association between HPV vaccination and thrombotic events (Arnheim-Dahlström et al., 2013, Scheller et al., 2014, Naleway et al., 2016, Yih et al., 2016). Several included studies were not designed as VTE-only investigations, but rather as broader post-licensure safety evaluations in which VTE was a prespecified adverse outcome. For example, Arnheim-Dahlström et al. evaluated 53 autoimmune, neurological, and venous thromboembolic outcomes in 997,585 adolescent girls and found no association with VTE (RR 0.86, 95% CI 0.55–1.36). Yoon et al. assessed 33 serious adverse events in 441,399 South Korean girls, with VTE included among cardiovascular outcomes, and likewise found no increased risk (adjusted rate ratio 0.62, 95% CI 0.22–1.70). More recently, Faksová et al. examined 56 unique outcomes after HPV9 vaccination in a nationwide Danish source cohort of 854,586 adolescents, including seven thromboembolic outcomes, and found no evidence of increased thromboembolic risk. Moreover, by specifically focusing on VTE and incorporating the most recent evidence, our study provides a more targeted and updated assessment of this safety concern.

Although only eight studies met the eligibility criteria, the underlying evidence base was large. Across the included studies, study populations exceeded 4.4 million participants, and more than 3.8 million HPV vaccine doses were reported. Importantly, early safety signals suggesting a possible positive association were based on very small numbers of cases and were not statistically robust. In Gee et al., the non-significant elevation observed in females aged 9–17 years was based on only five confirmed VTE cases, all of whom had established thrombotic risk factors. Passive-surveillance signals were also vulnerable to reporting bias, and many reported cases had pre-existing VTE risk factors. Subsequent chart-confirmed and self-controlled studies did not reproduce this signal, supporting the interpretation that the early observations were more likely explained by residual confounding, sparse events, and methodological limitations rather than a causal effect of HPV vaccination.

With the evolution of vaccination strategies, our study further explored the safety profile in males and the use of the 9-valent vaccine (HPV9). This is particularly important as vaccination programs expand to gender-neutral cohorts. Our analysis found no increased risk of VTE in male populations, which aligns with the results from Frisch et al. (2018). Similarly, regarding the transition from quadrivalent (HPV4) to nonavalent vaccines, we did not observe any increased risk associated with the higher valency. Although the number of studies in these subgroups is relatively small, these findings provide preliminary but reassuring evidence for the global rollout of HPV9 and male vaccination programs.

There are several potential mechanisms supporting our findings that HPV vaccination does not cause VTE. First, the biological nature of the vaccine differs fundamentally from platforms linked to thrombosis. Recent concerns about vaccine-induced thrombosis are specific to adenoviral vector vaccines (e.g., for COVID-19), which can induce anti-platelet factor 4 antibodies (Greinacher et al., 2021). In contrast, HPV vaccines are protein subunit vaccines composed of virus-like particles (VLPs) without viral DNA, which induce a humoral immune response rather than systemic inflammation or platelet activation (Schiller and Lowy, 2012, Stanley, 2010). Second, the “signals” in early observational studies were likely due to residual confounding. VTE risk in adolescents is strongly influenced by lifestyle factors, such as obesity and oral contraceptive use (Lidegaard et al., 2011). These factors may be particularly relevant in adolescents/young women and in individuals with predisposing conditions, potentially contributing to subgroup-specific signals observed in early surveillance analyses. The initiation of oral contraceptives often coincides with the age of HPV vaccination. If studies do not fully adjust for these factors, it may lead to a spurious association. Our *meta*-analysis included self-controlled case series designs that effectively control for such time-invariant confounders, thereby strengthening the reliability of our results.

There are several limitations in our study. First, the total number of studies included was relatively small (n = 8), which limited our ability to assess publication bias. Second, although the total sample size was large, VTE is a rare event, leading to wide confidence intervals in some subgroup analyses (especially for males and HPV9). Third, most included studies were conducted in high-income countries, which may limit the generalizability of our findings to other populations. Finally, potential unmeasured confounding factors in the original observational studies could still affect the results, although we prioritized adjusted estimates.

In conclusion, our *meta*-analysis suggests that HPV vaccination is not associated with an increased risk of VTE. The safety profile remains favorable across different sexes and vaccine types. These findings support the continued recommendation of HPV vaccination for the prevention of HPV-related cancers and help alleviate vaccine hesitancy related to safety concerns.

## 5 Authorship contributions

All authors YW, GA and CH made substantial contributions to (a) the conception and design of the study (YW, CH), or acquisition of data (YW, GA), or analysis (YW, GA); (b) interpretation of data (YW,GA) and drafting the article (YW,GA) or revising it critically for important intellectual content (YW, GA, CH); and (c) final approval of the version to be submitted (YW, GA, CH).

## CRediT authorship contribution statement

**Yushu Wang:** Writing – original draft, Visualization, Methodology, Investigation, Formal analysis, Data curation, Conceptualization. **Guangyu Ao:** Writing – original draft, Visualization, Validation, Methodology, Investigation, Formal analysis, Data curation. **Claude Hughes:** Writing – review & editing, Project administration, Conceptualization.
